# The therapeutic potential of epigenetic manipulation during infectious diseases

**DOI:** 10.1016/j.pharmthera.2016.07.013

**Published:** 2016-11

**Authors:** Joby Cole, Paul Morris, Mark J. Dickman, David H. Dockrell

**Affiliations:** a Department of Infection and Immunity, University of Sheffield Medical School, UK; b Sheffield Teaching Hospitals, UK; c Chemical and Biologic Engineering, University of Sheffield, UK

**Keywords:** Epigenetic, Chromatin, Immune regulation, AMPK, Adenosine monophosphate activated protein kinase, ART, Anti-retroviral therapies, BCG, Bacille Calmette–Guerin, BET, Bromodomain and extra terminal domain family of proteins, cagPAI, Cytotoxin-associated gene A pathogenicity island, C/EBPα, CCAAT/enhancer binding protein-α, Chip-seq, Chromatin immunoprecipitation and sequencing, EZH2, Enhancer of Zeste 2, H3, Histone H3, H3K4me1, Histone 3 lysine 4 monomethylation, H3K4me3, Histone 3 lysine 4 trimethylation, H3K23, Histone lysine 23, H3K27ac, Histone 3 lysine 27 acetylation, H3K9meSe10phosK14ac, Histone 3 lysine 9 methylation, serine 10 phosphorylation and lysine 14 acetylation, HKMT, Histone lysine methyltransferase inhibitors, H3S10, Histone 3 serine 10, H3T3, Histone 3 threonine 3, HDAC, Histone deacetylase, HDACi, Histone deacytelase inhibitor, HAT, Histone acetyl transferase, HIF-1α, Hypoxia-inducible factor, HSP70, Heat shock protein 70, HSPC, Hematopoietic stem cells, IFN, Interferon, IL-10, Interleukin 10, IL-12, Interleukin 12, IL-4, Interleukin 4, IRAK, Interleukin receptor-associated kinase, JMJD, Jumonji domain, LPS, Lipopolysaccharide, TLR, Toll-like receptor, miRNA, microRNA, mTOR, Mammalian target of rapamycin, MBD2, Methyl-CpG binding domain protein 2, NAD, Nicotinamide adenine dinucleotide, NF-κΒ, Nuclear factor-κΒ, NK, Natural killer cells, NLR, Nucleotide-binding oligomerization domain protein like receptors, NO, Nitric oxide, PAMPs, Pathogen associated microbial patterns, PRR, Pattern recognition receptor, PTMs, Histone post-translational modifications, RLR, Retinoic acid inducible gene 1 like receptors, RomA, Regulator of methylation A, ROR, Retinoic acid receptor-related orphan receptor, ROS, Reactive oxygen species, SAHA, Suberoylanilide hydroxamic, SET, Suvar3–9, enhancer-of-zeste, trithorax, SIRT, Silent mating type information regulator, TF, Transcription factor, Tfh, Follicular T helper cells, TGF-β, Transforming growth factor beta, TNF, Tumor necrosis factor, Treg, Regulatory T-cells, TSA, Trichostatin A

## Abstract

Epigenetic modifications are increasingly recognized as playing an important role in the pathogenesis of infectious diseases. They represent a critical mechanism regulating transcriptional profiles in the immune system that contributes to the cell-type and stimulus specificity of the transcriptional response. Recent data highlight how epigenetic changes impact macrophage functional responses and polarization, influencing the innate immune system through macrophage tolerance and training. In this review we will explore how post-translational modifications of histone tails influence immune function to specific infectious diseases. We will describe how these may influence outcome, highlighting examples derived from responses to acute bacterial pathogens, models of sepsis, maintenance of viral latency and HIV infection. We will discuss how emerging classes of pharmacological agents, developed for use in oncology and other settings, have been applied to models of infectious diseases and their potential to modulate key aspects of the immune response to bacterial infection and HIV therapy.

## 1 Transcriptional responses in immune cells

Host defense against infectious pathogens requires a coordinated immune response. Traditionally innate immunity has been viewed as generic and rapid, as lacking immunological memory and occurring with similar magnitude on rechallenge while adaptive immunity is more specific but requires time to mature. Innate immunity is mediated by pattern recognition receptors (PRR, such as Toll-like receptors (TLR), nucleotide-binding oligomerization domain protein like receptors (NLR) and retinoic acid inducible gene 1 like receptors (RLR), that recognize pathogen associated microbial patterns (PAMPs) and mediate an inducible response to micro-organisms (Yoneyama and Fujita, 2007, Chen et al., 2009). PRR and the resulting cytokine signals stimulate the production of transcription factors (TFs) that regulate signal transduction and effector responses. Functional specialization is reflected by subsets of monocytes (Schmidl et al., 2014) and different macrophage activation phenotypes (Mosser & Edwards, 2008).

Regulation of the inducible transcriptional program to pathogens includes post-transcriptional control (Medzhitov & Horng, 2009). Transcription of immune genes involves the interaction of promoter regions with distant enhancers that are brought into close spatial alignment through the looping out of interlinking DNA (Smale, 2010). These distant enhancers are particularly important as binding sites for tissue-specific TFs (Lee, Kim, Spilianakis, Fields, & Flavell, 2006). Several DNA-binding proteins act synergistically to integrate the activation of multiple signal transduction pathways and the generation of several distinct TFs (Smale, 2010). The resulting complex of DNA-binding proteins will ensure the acquisition of the chromatin remodeling factors, transcription co-activators, general TFs and RNA polymerase II to activate gene transcription. This results in a relative stimulus and cell specific response. However, a growing body of literature suggests that the configuration of the chromatin structure of immune genes and their regulatory elements is particularly important in determining the specificity of the immune response (Smale, Tarkhovsky, & Natoli, 2014). These chromatin changes are themselves directed in part by the influences of lineage and cell-type specific TFs during cell development, as exemplified by the macrophage responses to the developmental regulator PU-1 (Natoli, Ghisletti, & Barozzi, 2011). These cell-type specific influences then interact with environmental influences to regulate gene transcription in response to infection.

T-cell receptors and immunoglobulin are much more diverse than PRR (Robins et al., 2009, Rothenberg, 2014). The development of specific subsets, aids host defense (Luckheeram, Zhou, Verma, & Xia, 2012) and is regulated by specific TFs e.g. T-bet for T helper (Th) 1 (Lugo-Villarino, Maldonado-Lopez, Possemato, Penaranda, & Glimcher, 2003) and the retinoic acid receptor-related orphan receptor (ROR) γT and RORαΤ for Th17 (Yang et al., 2008). B-cells can also be separated into functional subsets with distinct transcriptional programs regulating their development (Allman & Pillai, 2008). Other lymphocyte subsets function as having features more typical of innate immune cells e.g. γδT-cells requiring the TF SOX13 for their development (Melichar et al., 2007). Unique TF profiles also define subsets of NK cells (Fu et al., 2014).

A key characteristic of both innate and adaptive effector functions is that functional subsets demonstrate significant plasticity, allowing a more flexible response to pathogens. Regulatory T-cells (Treg) can become Th17, while other conditions allow them to develop into follicular T helper cells (Tfh) (Xu et al., 2007, Tsuji et al., 2009). Th17 can become Th1 cells when exposed to IL-12 and Th2 cells in the presence of IL-4 (Bending et al., 2009, Lee et al., 2009).

## 2 Epigenetic regulation of transcription

Epigenetics is defined as a “ stably heritable phenotype resulting from changes in a chromosome without changes in the DNA sequence” (Berger, Kouzarides, Shiekhattar, & Shilatifard, 2009). The term epigenetic is, however, increasingly taken to include transient chromatin modifications as long as they result in altered gene transcription (Natoli, 2010). Epigenetic changes play a pivotal role in the adaptation of the transcriptional response (Jenuwein & Allis, 2001). Mechanisms include DNA methylation, histone post-translational modifications (PTMs), long non-coding RNA and microRNA.

Histone PTMs have been the subject of particularly intensive investigation and we will focus on these in this review since these dynamic changes allow modulation of the immune response to infection, even though these are not necessarily inheritable.

**Fig. 1:**
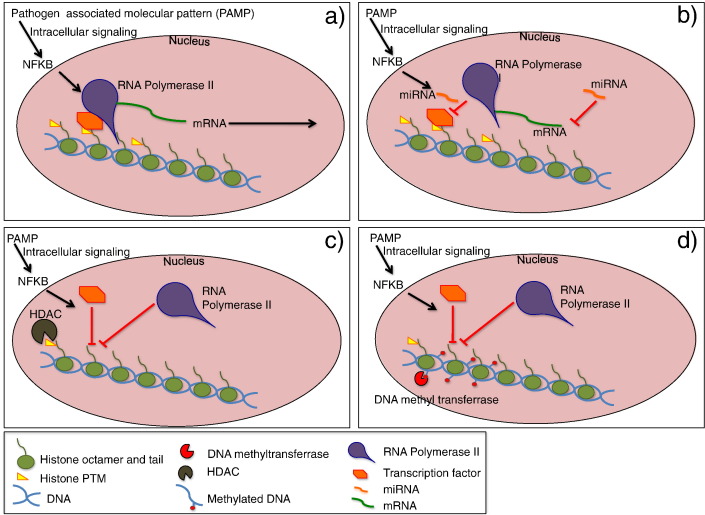
Schematic representation of the main epigenetic mechanisms. **a**) the presence of favorable PTM (such as acetylation) on the histone tail allows the binding of TFs in turn attracting RNA polymerase. **b**) This vignette illustrates the mode of action of microRNA inhibiting the binding of RNA polymerase and or the trafficking of mRNA. **c**) Illustrates the removal of PTMs by specialized enzymes (such as HDAC) in turn modifies the confirmation of the chromatin and inhibits the binding of TFs and RNA polymerase. **d**) In addition DNA methylation blocks the binding of TFs thereby inhibiting gene transcription from occurring.

Although these modifications may be transient they are more sustained than the transient protein PTMs observed with signaling molecules and thus allow a mechanism for extending the response period to external stimuli (Ivashkiv, 2013). Histone octamers composed of pairs of histone proteins H2A, H2B, H3 and H4 form nucleosomes (Olins & Olins, 2003). Histone PTMs refer to the chemical alteration predominantly of the N-terminal tail of the histones including, but not limited to, acetylation, methylation, phosphorylation, and ubiquitination. These chemical modifications control access of proteins to the underlying DNA or the terminal tail of the histones, and therefore regulate gene transcription (Shahbazian & Grunstein, 2007). The effect on gene transcription of a given PTM can vary, for example methylation of lysine or arginine residues can enhance or inhibit transcription depending on the residue modified and the degree of methylation (Kouzarides, 2007). These histone PTMs are regulated by families of enzymes which have the potential to be therapeutically targeted; histone acetyl transferases (HATs) and histone deacetylases (HDACs) regulate acetylation while lysine or arginine methyltransferases, lysine demethylases, arginine deaminases and arginine demethylases regulate methylation status (Kouzarides, 2007). The concept of the epigenetic landscape has been introduced to reflect the overall influence of DNA methylation status, histone PTMs and proteins pre-bound to promoter and enhancer regions on the accessibility for binding of classic signaling TFs like NF-κB (Ivashkiv, 2013). This mechanism allows gene transcription to respond to the environment, including stimuli from infection (Jenuwein & Allis, 2001) (summarized in Fig. 1). The histone response is therefore a dynamic sensor of the cell's environment. As such epigenetic manipulation makes for an attractive therapeutic target as it allows for a reversible modification in host gene expression.

## 3 Innate immunity

Many pathogens colonize the host prior to establishing invasive disease, as illustrated by extracellular bacteria (Kadioglu et al., 2008) but similar principles apply for fungi and parasites*.* The interactions between the innate immune system and the pathogen are a key factor in determining susceptibility to disease and likelihood of clinical infection (Dockrell, Whyte, & Mitchell, 2012). This is clearly dependent on how effective the transcriptional response of innate immune cells is, particularly macrophages as orchestrators of the innate immune response. These early responses are also important for intracellular pathogens such as viruses and bacteria.

### 3.1 Macrophage activation

Macrophages represent the cornerstone of the innate immune response in tissues (Twigg, 2004, Aberdein et al., 2013). Resident macrophages, originating from a fetal origin are supplemented by monocyte-derived macrophages recruited to sites of inflammation (Shi & Pamer, 2011). Macrophages have been described as either “classically” activated macrophages (M1 phenotype), that are particularly important for the immune response to intracellular bacteria, and generate increased levels of reactive oxygen species (ROS), nitric oxide (NO) (Dalton et al., 1993), or as “alternatively” activated macrophages (M2 phenotype) that play key roles in wound healing but also immunity to helminths and other parasites (Anthony et al., 2006) (Mosser & Edwards, 2008). In reality every stimulus results in a slightly different transcriptional profile (Murray et al., 2014) and activation states are highly plastic (Daigneault, Preston, Marriott, Whyte, & Dockrell, 2010). Given the different impact on disease processes modulation of the activation-associated transcriptional profile represents a potential therapeutic approach that can promote resolution of inflammation and tissue repair or increase pathogen clearance.

### 3.2 Epigenetic modification and macrophage differentiation

The differentiation processes driving monocytes to become macrophages or dendritic cells have been extensively studied (Saeed et al., 2014) and comprehensive review of the subject can be found (Álvarez-Errico, Vento-Tormo, Sieweke, & Ballestar, 2014). Myeloid differentiation is characterized by DNA hypomethylation, although it is dynamically regulated (Bocker et al., 2011). It also involves changes in histone PTMs and HDAC7, which represses macrophage specific genes, is repressed by the lineage specific TF CCAAT/enhancer binding protein-α (C/EBPα) that acts in concert with the PU-1 TF to promote macrophage differentiation (Barneda-Zahonero et al., 2013). Recently, mass spectrometry approaches have been utilized to identify histone PTMs occurring during the differentiation process from monocyte to either dendritic cell or macrophage. The results demonstrated that the macrophage differentiation process is associated with the combinatorial modification lysine 9 methylation, serine 10 phosphorylation and lysine 14 acetylation on histone H3 (H3K9meS10phosK14ac), whereas the differentiation to a dendritic cell was associated with acetylation of lysine 16 on histone H4 (Nicholas et al., 2014). This suggests that distinct histone PTMs occur during differentiation, in a cell-type specific manner. In addition genome wide studies show how the lineage TF PU-1 facilitates nucleosome remodeling and co-operates with other small subsets of lineage specific TFs to enable H3 lysine 4 monomethylation (H3K4me1) at a range of gene regulatory elements. These then act as beacon sites for the recruitment of further regulators that ultimately ensure the cell specific transcriptional response (Heinz et al., 2010).

These differences may allow differential regulation of signature inflammatory responses important in responses to pathogens. The exposure of immature macrophages to trichostatin A (TSA) (a class I and II HDACi) leads to increased global levels of H3 and H4 acetylations. This results in an increase in the release of the pro-inflammatory cytokine TNF-α. However, this effect is not seen in mature macrophages suggesting that this reversible chromatin modification and its capacity to influence TNF-α expression are only present during a fixed window of the maturation process (Lee, Kim, Sanford, & Sullivan, 2003). Thus the maturation process influences the cell's epigenetic profile and alters the ability of certain modifications to act as regulation points for cytokine responses. In this case monocytes, cells known to generate high level TNF-α responses (Daigneault et al., 2010), are equipped with the ability to regulate TNF-α responses by global reduction in both total H3 and H4 acetylation patterns but tissue macrophages which have less high output expression of TNF-α have lost this regulation check-point (Lee et al., 2003). These differentiation-dependent points of regulation involving histone PTMs may in turn be influenced by prior experience.

The relationship between histone PTMs and gene expression does not conclusively demonstrate directionality. This is illustrated by the case of differentiation and whether histone PTMs are a consequence of gene activation during differentiation or a key driver of the differentiation process has been debated.

Nevertheless the example of TSA influencing acetylation levels in monocytes prior to differentiation suggests that alterations of histone PTMs precede differentiation and influence the process (Lee et al., 2003). The epigenetic regulation of the processes governing differentiation of monocytes could therefore represent a target with which to influence macrophage phenotype and the microbicidal and inflammatory responses during infection.

### 3.3 Chromatin remodeling and macrophage transcriptional profiles

The different macrophage activation states have different transcriptional profiles (Ehrt et al., 2001) and different signatures of histone PTMs (De Santa et al., 2009, Satoh et al., 2010). In general gene loci associated with polarization state of macrophages may be in a repressed state, characterized by the presence of repressive marks such as H3 lysine 9 trimethylation (H3K9me3) and H3K27me3 and heterochromatin. In a poised state the abovementioned repressive marks are found in association with marks such as H3K4me3 and H3K9, 14-acetylation (ac) and the chromatin structure is partially open while in an active state the repressive marks are removed and active marks, such as H3K4me3, histone 3 serine 10 phosphorylation (H3S10phos) and histone 4 acetylation (H4ac) are present, allowing formation of euchromatin and gene transcription (Ivashkiv, 2013). In differentiated macrophages TFs such as PU-1 and C/EBPα open the regulatory regions. Even resting macrophages have acquired permissive marks on promoters and enhancers can be in a poised state. Basal transcription of pro-inflammatory cytokines (e.g. TNFα) occurs but is kept restrained without appropriate activation by mechanisms that include recruitment of HDACs and histone demethylases.

During the initiation of classical activation, the transcription start sites of genes associated with pro-inflammatory responses to lipopolysaccharide (LPS are found to have an increase in H3K4me3 at gene promoters, associated with increased gene transcription (De Santa et al., 2009). Histone modifications also poise gene enhancers to facilitate rapid activation following subsequent LPS stimulation (Smale et al., 2014). H3K4me3 and H3Kac have been associated with epigenetic changes at a range of cytokine promoters such as *Tnfa*, *Il6* and *Ifnb* (Ivashkiv, 2013). These changes contribute to the cell specificity of the response. The monomethylated lysine 4 on H3 (H3K4me1) mark may be a feature of enhancers in a poised state and is enriched in both inactive poised enhancers (where it occurs in the absence of histone acetylation) and active enhancers (where it occurs in the presence of histone acetylation) (Creyghton et al., 2010, Rada-Iglesias et al., 2011). The H3K4me1 mark also plays a role in latent enhancers (Ostuni et al., 2013). These transcriptional enhancers have been defined as lacking marks and TF binding at baseline in differentiated cells, acquiring the mark and TF binding following stimulation, requiring both stimulus dependent and lineage specific TFs, retaining the H3K4me1 mark after stimulation cessation and enhancing subsequent responses following secondary stimulation. The persistence of H3K4me1 as a stable mark has been demonstrated in a subset of interferon gamma (IFN-γ) regulated genes in macrophages and can enable innate memory through enhanced responses on re-stimulation.

Active enhancers frequently recruit p300, a protein that contains a histone acetyltransferase domain (Heintzman et al., 2009, Chen and Li, 2011). p300 is found bound to a large group of enhancers with the H3K4me1 mark, that are activated by LPS in murine bone-marrow derived macrophages (Ghisletti et al., 2010). These enhancers also contain binding sites for the lineage restricted TF PU-1, which is critical in maintaining the H3K4me1 mark. Stimulus specificity in macrophage transcriptional responses can involve TF co-operativity but chromatin remodeling also plays a role (Smale et al., 2014). LPS also stimulates de novo enhancers involving H3K4 methylation, mediated by the histone methyltransferases MLL1, 3 and 4 that work in co-operation with the TFs PU-1, C/EBPs and NF-κΒ (Kaikkonen et al., 2013). Histone acetylation appears to precede these H3K4 methylation events. Gene transcription following active stimulation also requires activation of enzymes that can remove repressive marks; LPS-induced induction of H3K4me3 results in preferential recruitment of the histone demethylase Jumonji domain-containing 3 (JMJD3) that in turns leads to loss of the repressive H3K27me3 mark (De Santa et al., 2009). There are many variations in how specific enzymes regulate the transcription of various genes; HDAC3 enhances IL-6 responses to LPS, and also regulates IFN-β (Chen et al., 2012), while its loss favors alternative activation in macrophages (Mullican et al., 2011). JMJD3 also facilitates removal of repressive marks on genes associated with alternative activation (Ishii et al., 2009, Satoh et al., 2010). However the increasing identification of specific genes that regulate the epigenetic events controlling the magnitude and kinetics of pro-inflammatory signaling offer the potential to modify these responses when excessive or inadequate through therapeutic targeting of this process.

Some transcriptional responses to LPS are associated with genes that have CpG island promoters and these genes have open chromatin in an unstimulated state that facilitates rapid but transient induction of the gene (Smale et al., 2014). Other LPS responses involve genes with promoters containing low CpG promoters. These genes usually have chromatin that appears in an inactive state in the unstimulated state and gene activation requires chromatin remodeling by SWItch/Sucrose Non-fermentable SWI/SNF nucleosome remodeling complexes (Smale et al., 2014). LPS responsive genes that require some chromatin remodeling by this mechanism include the *IL-6*, *IL-12b* and inducible nitric oxide synthase (*NOS2*) genes. These responses are associated with different kinetics of nuclear factor-κΒ (NF-κΒ) binding and more sustained and higher level transcription. As an example of this mechanism it has been shown that chromatin remodeling allows access of c-Rel-containing NF-κΒ to the *IL-12b* promoter (Weinmann et al., 2001).

The different macrophage polarization states are characterized by differences in their metabolic profile; LPS-stimulated macrophages activate glycolysis and suppress oxidative phosphorylation and mitochondrial respiration, and accumulate the tricarboxylic acid cycle intermediate succinate, which stabilizes the TF, hypoxia-inducible factor (HIF)-1α, leading to enhanced release of the pro-inflammatory cytokine IL-1β (Tannahill et al., 2013). This profile is also associated with decreased activation of silent mating type information regulator 2 homolog (SIRT) 5 (Tannahill et al., 2013), which acts as a deacetylase and a desuccinylase. This has the potential to modify histone PTMs involving acetylation and succinylation and alter chromatin modeling (Haigis and Sinclair, 2010, Park et al., 2013). The sirtuins are a family of highly conserved NAD dependent histone deacetylases and are metabolic sensors (Shih and Donmez, 2013, Papanicolaou et al., 2014). Sirtuin activity links diurnal variation in metabolism to inflammatory responses regulated by the circadian clock machinery (Verdin, 2014). As a mitochondrial sirtuin, SIRT 5, has the potential to link metabolic changes to chromatin remodeling. Monocytes stimulated with LPS or β-glucan were observed to have lower levels of oxygen consumption suggesting a shift to glycolysis. It was shown in macrophages that the control of these metabolic processes was in part regulated by epigenetic modifications involving loss of H3K27ac and increase in H3K4me3 at the promoter sites for genes involved in metabolic responses, in particular the mammalian target of rapamycin (mTOR) (Cheng et al., 2014). Moreover following challenge with β-glucan mice demonstrated HIF-1α-dependent protection against *Staphylococcus aureus*, associated with a shift to glycolysis and upregulation of JHDM1D ((Jumonji C domain-containing histone demethylase), illustrating another potential mechanism linking shifts in metabolism to epigenetic changes that enhance the innate immune response to infection (Cheng et al., 2014). It has been suggested that pro-inflammatory cells, such as M1 activated macrophages and Th17 cells are characterized by an increased reliance on glycolysis whereas cells with a more anti-inflammatory profile or resting cells such as the M2 macrophage, Treg and T memory cells are more reliant on oxidative metabolism (O'Neill & Hardie, 2013). Finally, acetylCoA derived from the metabolism of pyruvate is the principal acetyl donor for histone acetylation (Wellen et al., 2009).

### 3.4 Macrophage training and tolerance

The concept of trained immunity and tolerance has been debated but has emerged as a framework to explain the potential existence of innate immune memory. The process by which prior stimulation with β-glucan and BCG enhance responsiveness and protection to subsequent infectious challenge, has been termed trained immunity (Netea, Quintin, & van der Meer, 2011). Recent studies have linked innate immune memory to the formation of latent enhancers that are unmarked in the basal state and become marked following initial exposure to stimuli, but the persistence of one key mark, H3K4me1, at latent enhancers, has only been determined for relatively short time spans in vitro (Ostuni et al., 2013), so the long term impact of this mechanism remains to be determined*.*

Early observations showed increased survival from infection with *Babesia microti* in mice vaccinated with Bacille Calmette–Guerin (BCG) (Clark, Allison, & Cox, 1976). This observation of enhanced response following initial exposure has been seen in a number of different systems including plants, where it is known as systemic acquired resistance (Durrant & Dong, 2004). Recently, animal studies have shown that the mechanisms governing this enhanced response may involve epigenetic changes. Mice were found to be protected from subsequent re-exposure with lethal doses of *Candida albicans* (Quintin et al., 2012). The training required the β-glucan receptor dectin-1 and the Raf-1 pathway and was associated with increases in H3K4me3 (Quintin et al., 2012). Chromatin immunoprecipitation and sequencing (Chip-seq) analysis demonstrated that the H3K4me3 mark was associated with the promoters of genes involved in immune responses including dectin-1, C-type lectin receptors, pro-inflammatory cytokines such as TNF-α, IL-6 and IL-18, TLRs and the TLR adaptor protein Myd88. In studies of human subjects who had received BCG vaccination, macrophages were found to have increased TNF-α and IL-1β production when re-exposed to mycobateria or *C. albicans* (Kleinnijenhuis et al., 2012). This mechanism affords the host the ability to adapt to its environment and represents a form of innate immune memory resulting from chromatin remodeling. Although the duration of these changes is unclear, stem cell-derived macrophages, exposed to TLR2 agonists prior to differentiation, demonstrate lower levels of ROS production following subsequent stimulation (Yáñez et al., 2013), suggesting that epigenetic changes in macrophages may be sustained for prolonged periods. Other innate immune cells such as NK cells can also develop sustained immunological memory following sensitization to mediate contact hypersensitivity in mice (O'Leary, Goodarzi, Drayton, & von Andrian, 2006). Moreover, BCG vaccination was shown to lead to increased pro-inflammatory cytokine production (IL-1β, IL-6 and TNF-α) when NK cells were stimulated ex vivo (Kleinnijenhuis et al., 2014).

In contrast to a heightened response associated with training, it has long been established that macrophages can become tolerant following LPS challenge, leading to a decreased responsiveness to LPS and other stimuli on rechallenge. Clinically this phenotype is demonstrated by the immune paralysis associated with critical illness and sepsis. Mice pre-treated with LPS demonstrated increased survival in a polymicrobial model of sepsis caused by cecal ligation and puncture (Wheeler et al., 2008). Tolerance can be induced by repeated exposure to TLR4 agonists such as LPS, whereas the response to other stimuli induces the enhanced pro-inflammatory response seen in trained immunity (Ifrim et al., 2014). Tolerance is also associated with specific changes in the epigenetic state of cells in particular decreased H4ac (Foster, Hargreaves, & Medzhitov, 2007). The non-specific innate immune memory provided by training and tolerance can be viewed as an adaptation to help ensure inflammatory responses to pathogens are appropriate but not excessive. Tolerance helps decrease the risk of tissue injury and death from an exaggerated immune response to infection. As such the manipulation of these states represent a potential therapeutic approach to limit excessive inflammation and potentially improve bacterial clearance from a previously stimulated macrophage. A more recent analysis has compared the histone marks associated with differentiation of macrophages from monocytes after the development of β-glucan induced training or LPS-induced tolerance (Saeed et al., 2014). Dynamic changes in H3K27ac in training and tolerance with a subset of responses that were unique to tolerance were observed. Sites of histone methylation were more constant and marked promoters or enhancers were susceptible to dynamic acetylation at H3K27. The transcription profile of tolerance was associated with modules typically seen in monocytes but not naïve macrophages, including surprisingly some pro-inflammatory genes, but it also included transcription of some negative regulators of inflammatory responses such as IL-1 receptor-associated kinase 3 (IRAK3), a negative regulator of TLR signaling. Training resulted in upregulation of molecules seen in naïve macrophages, including those involved in metabolism.

A separate phenomenon that appears distinct from tolerance is seen when immune cells are rechallenged with certain toxins. This phenomenon, which has been described for anthrax lethal toxin, is termed toxin-induced resistance. When macrophages are exposed to a sub-lethal dose of the toxin they are then resistant to the cytolytic effects of higher potentially lethal doses on rechallenge. A small proportion of macrophages (2–4%) can retain this resistance for 5–6 weeks and the mechanism of this phenomenon appears to involve HDAC8 (Ha, Han, Reid, & Kim, 2014). HDAC8 reduced H3 lysine 27 acetylation. This suggests that modulating enzymes regulating epigenetic processes could also influence the response to microbial toxins.

Overall a wealth of information on the role of epigenetic responses in regulating innate responses, particularly in macrophages is emerging. However, if these are to be exploited therapeutically clarification is still required on how long the changes documented in vitro last in vivo, clarification of which enzymes regulating histone PTMs have the degree of specificity to allow their selective modulation and finally identification of which processes have significant enough functional consequences to justify their targeting.

## 4 Epigenetic changes and adaptive immune responses

The regulation of the adaptive immune system also involves epigenetic modifications (Cuddapah, Barski, & Zhao, 2010). Lymphocytes are poised to respond to antigen recognition with a transcriptional program that involves genes, regulating metabolism, proliferation and clonal expansion. The epigenetic landscape is essential for lymphocyte subset specialization while metabolic regulation is critical to the development of immunological memory (Chang, Wherry, & Goldrath, 2014). Resting naïve T-cells have low levels of glycolysis, but after encountering a foreign antigen the differentiating T-cell increases its reliance on aerobic glycolysis, (DeBerardinis, Lum, Hatzivassiliou, & Thompson, 2008). Both the AMP activated protein kinase (AMPK) and mTOR are the key in controlling T-cell metabolic activity (Michalek et al., 2011).

Th1 cells are required to control intracellular pathogens, Th2 cells to respond to parasites and Th17 cells for antibacterial roles at mucosal surfaces (Kanno, Vahedi, HiraHara, Singleton, & O'Shea, 2012). It has been shown that these different lineages are associated with different acetylation status at of the promoter regions of *IL-4* and *IFN-γ* genes (Messi et al., 2003). Moreover, the promoter regions of certain genes such as *IL-4* and *IFN-γ* are associated with H3K4me3 and H3K27me3 in a lineage specific manner (Wei et al., 2009).

CD8^+^ T-cells provide one of the best examples of epigenetic regulation in the immune systems. *Cd4* gene silencing involves epigenetic mechanisms since it persists even after deletion of the transcriptional silencer during thymocyte development (Zou et al., 2001, Tanuchi et al., 2002). During the development of memory CD8^+^ T-cells, there is an increase in the levels of acetylation on H3K9 and K14 (DiSpirito & Shen, 2010). This results in a number of genes being poised for translation allowing a faster response on re-exposure. Furthermore, the rapidly inducible genes of poised lymphocytes are associated with H3 acetylation and H3K4me3 modifications in their promoter regions (Roh, Cuddapah, Cui, & Zhao, 2006). Thus there are theoretical opportunities to modify immune responses to pathogens and vaccines through epigenetic manipulation of lymphocyte subsets.

## 5 Epigenetics and non-viral infections

The importance of epigenetic changes occurring as part of the pathogenesis of infectious diseases is becoming increasingly understood (Bierne, Hamon, & Cossart, 2012). A range of micro-organisms induce epigenetic modifications during infection (Table 1). These include *Mycobacterium tuberculosis* (Yaseen, Kaur, Nandicoori, & Khosla, 2015), *Shigella flexneri* (Harouz et al., 2014) and the rickettsial pathogen *Anaplasma phagocytophilum* (Garcia-Garcia, Barat, Trembley, & Dumler, 2009).

Changes in histone PTM, DNA methylation and miRNAs all play a role in the response to infection. Indeed miRNAs are induced following exposure to LPS (Taganov, Boldin, Chang, & Baltimore, 2006) and by a range of different bacterial pathogens including *Listeria monocytogenes* (Schnitger et al., 2011), *Helicobacter pylori* (Zhang et al., 2008) and *M. tuberculosis* (Rajaram et al., 2011). The role of miRNAs in response to infection has recently been reviewed (Staedel and Darfeuille, 2013, Maudet et al., 2014) and as our focus has been on discussion of histone PTMs and their therapeutic potential we will not discuss these further. Furthermore, sites of DNA methylation have also been shown to be actively modified during infection (Pacis et al., 2015). Alterations in DNA methylation profiles can be identified following infections with agents such as *M. tuberculosis* and differences can be observed in patients with latent, as opposed to active TB, raising the possibility that they could be used as biomarkers of infection (Esterhuyse et al., 2015).

Epigenetic mechanisms can also be employed by pathogens to regulate their gene transcription. DNA methylation varies between *Salmonella enterica* serovars and may influence virulence (Pirone-Davies et al., 2015). HDACs modulate the transcriptional responses of *Plasmodium falciparum* (Andrews, Tran, & Fairlie, 2012) and another protozoan *Entamoeba histolytica* employs H3 lysine 27 dimethylation (H3K27me2) as a repressive mark to enable transcriptional gene silencing (Foda & Singh, 2015).

### 5.1 Epigenetics and bacteria

Examples of pathogens altering host gene transcription by modifying the host cell's epigenome via modulation of histone PTM have been provided by *Legionella pneumophilia*, *H. pylori* and *L. monocytogenes* (Hamon et al., 2007, Ding et al., 2010, Rolando et al., 2013). When gastric epithelial cells were infected with *H. pylori,* a time dependent dephosphorylation of H3S10 was observed as well as a decrease in H3K23ac (Ding et al., 2010). Production of IL-8 in response to *H. pylori* of these epithelial cells was reduced by TSA, suggesting that it involved histone deacetylation events. Interestingly, this mechanism is likely to be both organism and cell specific since in THP-1 cells the dephosphorylation of H3S10 was associated with the IL-6 promoter and resulted in an increase in IL-6 transcription (Pathak et al., 2006). The exact mechanism by which *H. pylori* causes alterations in histone PTM change is not fully understood, although it is apparent that it involves the cytotoxin-associated gene A pathogenicity island (cagPAI), since deletion of this but not cagA or other factors induced the dephosphorylation of H3S10 (Ding et al., 2010). These changes were also associated with upregulation of the oncogene c-Jun and with downregulation of heat shock protein (hsp) 70, showing that they not only contribute to inflammation but potentially also tumor development, which in the case of hsp70 the authors suggested was related to reduced protection against stress-induced protein denaturation and aggregation. Fehri et al. showed that *H. pylori* infection resulted in dephosphorylation of H3S10 but also threonine at H3T3 in gastric epithelial cells (Fehri et al., 2009). These events were also linked to the cagPAI and a functional type 4 secretion system and were reversed by a DcagL mutant. The changes were associated with bacterial induced reduction of cell division cycle 25 (CDC25C) phosphatase and a resultant reduction in activation of the H3 kinase vaccinia-related kinase (VRK) I, which resulted in *H. pylori* induced pre-mitotic arrest.

In the case of *L. pneumophilia* the bacterium uses a type 4 secretion system effector, regulator of methylation A (RomA), a Suvar3–9, enhancer-of-zeste, Trithorax (SET) domain-containing methyltransferase, which causes H3K14me3 of the host cell and reduces H3K14ac (Rolando et al., 2013). This switches off gene transcription. The SET protein domain is 130 amino acids long, was first identified in *Drosophila*, and has been found in all eukaryotic organisms studied. All histone methyltransferases contain a SET domain apart from those of the DOT1 family (Dillon, Zhang, Trievel, & Cheng, 2005). Chip-seq data in the study by Rollando et al. established that the switch to the repressive mark H3K14me3 was associated with the promoter sites of genes involved in immune responses, in particular cytokines such as TNF-α and IL-6 and PRR such as TLR5, which responds to flagellin, and the Nod-like receptor Nalp 3 (Rolando et al., 2013). This provides support to the theory that pathogens including bacteria and viruses can use chromatin remodeling strategies to turn off immune responses. As such this process of bacterial manipulation of the host gene expression represents an interesting therapeutic approach as it would be possible to either target RomA specifically to prevent the trimethylation of H3K4 or use histone acetyl transferase or CRISPR-Cas9 (as discussed below) to inhibit removal of the acetyl group.

A further example of epigenetic modulation is provided by *L. monocytogenes,* that secretes the surface protein internalin B (InlB), resulting in translocation of a host cell class 3 HDAC, sirtuin 2 (SIRT2), to the nucleus. In this location SIRT2 deacetylates H3K18ac. This is associated with a decrease in expression of genes involved in DNA-binding and immune responses (Eskandarian et al., 2013). Moreover, *L. monocytogenes* also secretes listeriolysin O (a pore-forming cytolysin), which causes dephosphorylation of H3S10 and decreases the level of H4ac (Hamon et al., 2007). These responses occurred by a pore-forming independent mechanism. This correlates with a change in the transcriptional profile of HeLa cells, resulting in a decrease in genes involved in innate immune responses, including the neutrophil chemokine CXCL2 and DUSP4 a phosphatase involved in regulating MAPK signaling.

*A. phagocytophilum* survives within neutrophils and their myeloid precursors and has been shown to induce epigenetic changes in host cells (Garcia-Garcia et al., 2009). Genes encoding antimicrobial peptides and both enzymatic and oxidative host defense molecules are downregulated in myeloid cell lines (neutrophilic and monocytic) and these changes are associated with reduction in H3 acetylation patterns and upregulation of HDAC1 and HDAC2. Genetic and pharmacological approaches linked activity of HDAC1 to the altered expression of host defense genes, suggesting the potential to modulate infection through selective HDAC inhibition. This epigenetic mechanism involves Ankyrin A, a type IV secretion system expressed by *A. phagocytophilum* that interacts directly with HDAC1 (Rennoll-Bankert, Garcia-Garcia, Sinclair, & Dumler, 2015).

The intracellular survival of *M. tuberculosis* involves reduced responses to IFN-γ and an epigenetic contribution to this process was suggested in the monocytic THP-1 cell line (Wang, Curry, Zwilling, & Lafuse, 2005). HDAC inhibition with butyric acid or MS-275 reversed the ability of *M. tuberculosis* or *Mycobacterium avium* to block expression of HLA-DRα or HLA-DRβ mRNA, IFN-γ responsive transcripts, while mycobacterial infection inhibited IFN-γ induced histone acetylation. Although transcription of HDAC1–3 was not altered by *M. avium,* the authors demonstrated that mSin3A, which is present in a multi-component complex with HDAC1 and HDAC2, and is a functional co-repressor in HDAC-mediated inhibition of gene transcription, was upregulated. This provided evidence that mycobacteria can modulate IFN-γ signaling through epigenetic mechanisms in addition to established mechanisms, such as modulation of receptor expression or signal transduction.

Other bacteria also modulate histone PTMs. In a model of murine mastitis strains of *S. aureus* induced differing levels of H3K9 and H3K14 acetylations, as well as differences in microRNA responses, and those inducing higher levels of acetylation were associated with greater inflammatory responses and pathogen clearance (Modak et al., 2014). The same group also suggested that these histone marks influenced the inflammatory response to *Escherichia coli* in the same murine mastitis model (Modak et al., 2012).

It remains unclear what the consequences of epigenetic changes during acute bacterial infections are. They can enable pathogen subversion of the host immune response, but may also be used by the host to modify transcriptional profiles, for example to limit the damage induced by sustained inflammation or to develop the trained response to improve outcomes on re-exposure. If these consequences are better delineated these processes could represent attractive therapeutic targets in the future.

### 5.2 Epigenetics in sepsis

Sepsis defines a physiological state that results from a severe inflammatory response most often secondary to infectious process. It has been responsible for 2 to 11% of all intensive care unit bed occupancy and is associated with between 25 and 80% mortality (Angus & Wax, 2001). The initial response to sepsis is pro-inflammatory with release of pro-inflammatory cytokines predisposing to organ failure. This is driven by the recognition of PAMPs or damage-associated molecular patterns interacting with TLR or other PRR, leading to activation of NF-κΒ dependent genes and release of a variety of pro-inflammatory cytokines (Foster & Medzhitov, 2009). The most well studied trigger of sepsis is the response to LPS. It has been shown to lead to alterations in gene expression mediated via epigenetic modifications (Foster et al., 2007). This results in responses such as macrophage tolerance, which, as described above, can have harmful effects through the associated immune paralysis. Therefore epigenetic responses to sepsis represent an interesting therapeutic target.

### 5.3 Therapeutic targeting with histone deacetylase inhibitors in sepsis

There has been an increasing interest in the use of HDACi in sepsis. However, their impact is unclear as they have been associated with both promising survival benefits but also impaired bacterial clearance. When treating macrophages with broad spectrum HDACi, such as suberoylandilide hydroxamic acid (SAHA) and TSA, macrophage's microbial killing ability was impaired (Mombelli et al., 2011, Roger et al., 2011). The macrophages exposed to HDACi had decreased levels of ROS and NO, which impeded their microbiocidal responses to *E. coli* and *S. aureus*. On the other hand, in mice the use of the HDACi suberoylanilide hydroxamic acid (SAHA) is associated with clear survival benefits following cecal ligation and puncture (Li et al., 2010). Similar results were seen in mice treated with Tubustatin (a HDAC 6 inhibitor), which was associated with enhanced resolution in bacteraemia, less organ dysfunction and a modulated stress response (Zhao et al., 2014). This was associated with increased monocyte counts, reversal of lymphopenia and increased neutrophil counts. Nevertheless, the functional significance of the association with monocyte counts requires further clarification. Mice which received Tubustatin during a hemorrhagic shock model were subsequently protected from sepsis following cecal ligation and puncture in a two hit model of sepsis (Cheng et al., 2015). Furthermore, mice treated with the SIRT1 and 2 inhibitor, Cambinol, were protected from endotoxic and toxic shock (Lugrin et al., 2013).

Tubustatin enhanced macrophage generation of mitochondrial ROS (mROS), a TLR generated microbicidal response in human macrophages and increased intracellular killing of *E. coli* and *Salmonella typhimurium* (Ariffin et al., 2015). Broad spectrum HDACi such as SAHA and TSA also enhanced killing if administered at the time of bacterial challenge but if incubated with macrophages for 18 h before bacterial challenge, they reduced phagocytosis and overall impaired bacterial killing. A HDAC1–3 inhibitor had no effect on bacterial killing, suggesting that specific HDAC6 inhibition may be an important component of the modulation of sepsis as shown by the clearance of bacteremia and improved outcomes in mice, as shown by Zhao et al. (2014). This suggests that more specific targeting of enzymes that regulate epigenetic changes may enable improved responses while preserving key microbicidal responses.

## 6 Epigenetics in viral infection

Epigenetic changes are also seen in viral infections involving viruses such as human adenovirus (Horwitz et al., 2008), influenza A (Marazzi et al., 2012) and HIV (Han et al., 2012). The host cell restriction on the replication of endogenous retroviruses also involves epigenetic mechanisms. Recent evidence suggests an important role for the histone methyltransferase SET-domain bifurcated 1 (SETB1) in ensuring H3K9me3 and repression of endogenous retrovirus replication in differentiated B-cells from adult mice (Collins, Kyle, Egawa, Shinkai, & Oltz, 2015).

Epigenetic modifications regulate the balance between viral latency and replication as seen with herpes viruses (Kumar & Herbein, 2014). A proteomics approach revealed that CMV replication in primary fibroblasts resulted in several histone PTMs including increases in H3 lysine 79 dimethylation (H3K79me2) (O'Connor et al., 2014). This histone PTM was associated with upregulation of its methyltransferase, disruptor of telomeric silencing 1 like H3 lysine 79 methyltransferase (DOT1L) and knockdown of DOT1L markedly reduced CMV replication. CMV replication was also associated with an increase in H3 lysine 27 methylation/histone 3 lysine 36 dimethylation (H3K27meH3K36me2) and a decrease in histone 4 lysine 16 acetylation (H4K16ac). During human herpesvirus-8 infection SIRT1 helps maintain latency by promoting the repressive mark H3 lysine 27 trimethylation (H3K27me3) at the viral replication and transcription activator (He & Gao, 2014). SIRT1 knockdown or chemical inhibition promoted lytic infection with reduction of H3K27me3 and increases in the active H3K4me3 mark. Enhanced H3K4me3 is also a feature of lytic infection with herpes simplex virus where barrier to auto-integration factor 1 (BAF/BANF1) a factor bridging chromosomes to the nuclear lamina was found to play a role recruiting the histone methyltransferase (SETD1A) to immediate-early and early gene promoters in the virus, facilitating replication (Oh et al., 2015). Therefore a picture is emerging of enzymes that regulate histone PTMs at the site of viral gene promoters and either allow or repress viral gene transcription.

### 6.1 Human immunodeficiency virus-1

HIV-1 is a single-stranded RNA virus, which replicates by reverse transcription resulting in generation of a DNA duplex that integrates into the host genome in CD4^+^ cells, mainly CD4^+^ T-lymphocytes (Nisole & Saïb, 2004). Replication of the virus is dependent on host cell-derived TFs and while activation enhances replication in some cells, in long lived memory cells the HIV-1 virus can remain in a latent state of relative transcriptional quiescence.

Anti-retroviral therapies (ARTs) aim to block replication principally targeting key enzymes involved in reverse transcription, integration and maturation of the HIV-1 proteins by the HIV-derived protease. Therapeutic approaches also target steps involved in the entry process, involving binding to the chemokine receptor CCR5 and fusion to the plasma membrane (Arts & Hazuda, 2012). All existing approaches involve life-long therapy and a therapeutic strategy that targets the latent reservoir will be required if a therapeutic approach is to achieve a cure (Deeks et al., 2012b). In view of the role of epigenetic mechanisms in regulating viral latency discussed above modulating latency through targeting of epigenetic regulation is perceived to be a vital component of a strategy to deliver HIV cure.

### 6.2 Targeting the latent reservoir in human immunodeficiency virus-1 infection

The latent HIV-1 reservoir persists within resting memory CD4^+^ T-cells despite HAART (Siliciano et al., 2003). Reactivation of these resting cells has been suggested as a central mechanism of HIV-1 persistence, through viral transmission to uninfected activated CD4^+^ T-cells, despite sustained ART (Chun et al., 2005). Epigenetic transcriptional interference, via deacetylation and methylation of histones, antagonizes binding of DNA polymerase and hence also restrict generation of HIV-1 transcripts from the pro-viral DNA. The 5′ long terminal repeat (5′ LTR) sequence of HIV DNA is bookended by nucleosomes Nuc-0 and Nuc-1, offering potent sites for inhibitory modification of transcription. In particular, Nuc-1 is located in proximity to the transcription start site (Verdin, Paras, & Van Lint, 1993). The chromatin and histones associated with the 5′ LTR nucleosomes are subject to deacetylation and trimethylation modification by HDACs (Keedy et al., 2009) and histone methyltransferases (HMTs) (Friedman et al., 2011) respectively. Epigenetic modification by pro-viral DNA methylation has also been shown to exert an influence on latency in vitro (Blazkova et al., 2009) but has not yet been shown in a clinical context (Blazkova et al., 2012a).

Manipulating histone PTMs to facilitate viral replication is one component of the strategy termed ‘Shock and Kill’, with reversal of latency though therapeutic modulation of epigenetic transcriptional silencing and killing of the infected cells achieved by viral induced cell cytotoxicity or alternatively by the immune response to viral proteins produced during viral replication (Deeks, 2012a). There is no current answer to how much pro-viral activation is necessary to achieve cell death of the latently infected cells (Archin, Sung, Garrido, Soriano-Sarabia, & Margolis, 2014b). Future approaches must achieve controlled levels of reactivation of latency without harmful immune activation.

### 6.3 Therapeutic manipulation of histone post-translational modifications in human immunodeficiency virus therapy

Recognition of the role of histone PTMs in mediating HIV-1 latency provided several potential therapeutic targets. The weak HDACi valproic acid has been shown to increase HIV-1 transcription from pro-viral DNA and virus production in vitro from cultured cell lines with latent HIV-1 infection (Moog et al., 1996, Witvrouw et al., 1997). However, when administered to individuals with sustained suppression of viral replication, the majority of individuals did not achieve a significant reduction in the latent reservoir of CD4^+^ T-cells (Archin et al., 2008).

SAHA (vorinostat), like valproic acid has also enhanced HIV-1 replication (Archin et al., 2009a). Vorinostat targets several classes of HDACs, including class I HDACs. This group of HDACs inhibits transcription, through deacetylation of the lysine tail of core histones, so that access to DNA by TFs is antagonized, as well as by recruiting additional transcription-repressing histone-modifying complexes (Archin, et al., 2009b). A clinical trial identified sixteen aviremic (HIV viral load < 50 copies/ml) patients of whom eleven had evidence of vorinostat-induced increases in HIV RNA in purified resting memory CD4^+^ T-cells isolated by leukapheresis. Of these, eight patients stable on ART received an initial dose of 200 mg vorinostat to establish safety, and subsequently a single 400 mg dose (Archin et al., 2012). Total cellular H3 acetylation increased (median 1.6-fold, p < 0.01) in resting CD4^+^ T-cells isolated 6 h after drug exposure, in which HIV RNA expression also increased by a mean of 4.8-fold (range 1.5–10, p < 0.01). However, a diminishing response to successive vorinostat treatment was observed when administered on three successive days over eight weekly cycles (Archin et al., 2014a). Importantly, vorinostat was well tolerated by all with no adverse events reported.

The proof of concept that HDACi can increase viral replication from latent reservoirs has spurred experimentation with alternative and more selective HDACi. Antagonism of class I HDACs induced the desired acetylation of LTR-associated histones, whereas antagonism of class II HDACs had no significant effect (Archin, et al., 2009b). A number of alternative inhibitors with activity against class I HDACs, namely panobinostat, givinostat and belinostat, are in development as oncology therapies and may offer greater potency in vivo than vorinostat (Rasmussen et al., 2013). Panobinostat is a pan-HDAC inhibitor, has activity against classes I, II and IV HDACs (Prince & Bishton, 2009). In vitro it induced HIV RNA expression in latently infected cell lines (Rasmussen et al., 2013). A phase I/II trial, CLEAR, was recently completed (Rasmussen et al., 2014). Volunteers received 20 mg panobinostat three times per week every other week. Intracellular HIV RNA and DNA expression significantly increased, alongside detectable increases in HIV peripheral copy number in the blood. However, estimates of the number of cells with latent infection remained unchanged, suggesting limitations with the HDAC inhibitor strategy for reduction of the HIV-1 reservoir as currently employed.

The epigenetic mechanism by which post-integration pro-viral latency develops could be targeted in combination with approaches to increase TF availability. Activation of cytoplasmic NF-κΒ by the phorbol ester prostratin stimulates its translocation to the nucleus, where it activates HIV transcription (Williams et al., 2004). Valproic acid or vorinostat used in combination with prostratin displayed synergistic activation of latent provirus (Reuse et al., 2009). These in vitro models of latently infected cells demonstrated HDACi increased prostratin-dependent effects on the NF-κΒ binding capacity, promoting activation of the 5′ LTR of HIV and transcription of virus.

Histone lysine methyltransferase (HKMT) inhibitors have also been investigated as a potential therapeutic option. Methylated H3 has been demonstrated to be dimethylated (Imai, Togami, & Okamoto, 2010) and trimethylated (Pearson et al., 2008) at specific locations that interact with latent HIV-1 genomes, and have been considered repressive in other epigenetic settings (Kouzarides, 2002). Knockdown of the HKMT G9a, responsible for the dimethylation of H3 lysine 9 (H3K9me2), and knockdown of the HKMT enhancer of Zeste 2 (EZH2) permits increased accessibility of the HIV 5′ LTR, allowing viral transcription (Imai et al., 2010, Friedman et al., 2011). EZH2 is a subunit of the polycomb repressive complex 2 (PRC2) and mediates gene silencing through PTM of histones. It is required for the enzymatic function that induces the trimethylation of H3 lysine 23 (H3K27me3). The specific HKMT G9a antagonist BIX01294 enhanced HIV-1 gene expression when applied to latently infected T-cells in vitro and CHIP-seq identified G9a and H3K9me2 in the vicinity of the HIV-1 promoter region (Imai et al., 2010). In vitro assays in latently infected ACH-2 cells also revealed synergy between BIX01294 and vorinostat, increasing HIV-1 gene expression individually from 4.9-fold and 13.5-fold respectively to 47.1-fold when in combination. Furthermore, the combination of BIX01294 and the DNA methylation inhibitor 5-aza-2′-deoxycytidine (aza-CdR), which individually increased viral replication by 7.0-fold and 8.5-fold respectively, resulted in an increase in viral transcription to 29.6-fold (Imai et al., 2010). A pan-HKMT antagonist, 3-deazaneplanocin-A (DZNep) has been shown to be superior to BIX01294 in subsequent in vitro experiments utilizing a latently infected E4 cell line. E4 cells exposed overnight to BIX01294 and DZNep induced pro-viral expression in 21.1% and 31.5% of cells respectively (Friedman et al., 2011). Synergy between DZNep and vorinostat was also displayed after 48 h in experiments using 5 μM DZNep application followed by 0.5 μM vorinostat, with 15.3% and 4.2% respective inductions of latent viral expression when the agents were used individually compared to 29.3% when in combination (Friedman et al., 2011).

Targeting DNA methylation might represent a further epigenetic approach to target HIV latency on the basis of in vitro experiments. CpG hypermethylation of the 5′ LTR promoter and enhancer sequences confers transcriptional silencing that contributes to the establishment of the stable latent reservoir and develops as a late event when compared to transcriptional interference and chromatin modification (Blazkova et al., 2009). A cDNA library screen identified methyl-CpG binding domain protein 2 (MBD2) as a factor promoting HIV-1 latency (Kauder, Bosque, Lindqvist, Planelles, & Verdin, 2009). Inhibition of cytosine methylation with the DNA methylation inhibitor 5-aza-2′-deoxycytidine (aza-CdR) in vitro reduced recruitment of MBD2 and HDAC2 to an immortalized CD4^+^ T-cell, which is latently infected with the complete HIV-1 genome that is transcriptionally inhibited by hypermethylation at the promoter region. When used in combination, aza-CdR plus the NF-κΒ activator prostratin, stimulated greater HIV-1 expression in the immortalized CD4^+^ T-cell, compared to prostratin alone (Kauder et al., 2009). The combination of aza-CdR with vorinostat demonstrated weak synergism, but independent application of aza-CdR showed no increase in HIV-1 expression. The authors were also able to demonstrate methylation of HIV-1 CpG islands and latency in primary memory CD4^+^ T-cells. However, others have suggested latently infected resting CD4^+^ cells isolated from patients fully suppressed with ART only rarely display hypermethylation of the 5′ LTR sequence, questioning the potential utility of methylation inhibitors (Blazkova et al., 2012b).

## 7 Future drug targets and potential

As discussed above, there have been a number of different pharmacological compounds used in vitro and in vivo to modulate the epigenome. These range from HDACi, bromodomain inhibitors (Heerboth et al., 2014) and novel CRISPR/Cas9 coupled methyltransferases and acetylases (discussed below) (Hilton et al., 2015). HDACis have been used more extensively to date (see Table 2 for use in HIV trials), indeed several of them are now licensed for use in cutaneous T-cell lymphoma and multiple myeloma. There are 18 different kinds of HDACis in humans, which are divided into 4 classes based on their homology with yeast HDACis. However, as alluded to earlier, the impact of HDACis on innate immune function is mixed, they both help with the resolution of inflammation but also impair the microbiocidal functions of macrophages in some settings. Moreover, most of these studies have used pan-HDACis that have many off target effects and it is likely that the more specific novel treatments will allow for improved targeting. Strategies targeting site specific histone PTMs such as the Jumonji H3K27 demethylase inhibitor (GSK-J1/J4) which was shown to modify the post-LPS inflammatory response (Kruidenier et al., 2012), may allow these problems to be circumvented by decreasing the off target effects. Indeed HDACs have a diverse number of target proteins including structural proteins such as α-tubulin, DNA-binding nuclear receptors, transcription regulators and signaling mediators, both inside the nucleus but also in the cytoplasm. Furthermore, HDACs can also exert their activity on other HDACs as part of larger multiprotein complexes (Falkenberg & Johnstone, 2014); hence it is difficult to distinguish the specific site of action of broad HDACis. Most of the older HDACis, such as SAHA or TSA, target several different HDACs. Therefore the exact biological consequences are hard to untangle. More recently, isoform selective HDACis have been developed such as HDAC 6 inhibitor Tubastatin A (Vishwakarma et al., 2013). This may explain the mixed results of HDACis in sepsis as earlier trials used the less selective compounds inhibiting both classes I and II HDACs whereas the HDAC6 inhibitors had more positive results. Indeed as further compounds with greater selectivity are developed this will allow greater manipulation of individual epigenetic pathways.

The bromodomain inhibitors may also allow specificity in targeting and allow for individual histone PTMs to be manipulated in a dynamic fashion during the course of infection or inflammation. The bromodomain and extra terminal domain family of proteins (BET) act as adaptors that link histone acetylation status to transcription by incompletely defined mechanisms (Smale et al., 2014). However, the majority of all the BET proteins seem to act by a mechanism that involves the RNA elongation complex polymerase associated factor 1 complex that plays a role in RNA initiation and also elongation (Dawson et al., 2011). The ET domain of the BET proteins interacts with various proteins likely to influence chromatin remodeling including NSD3 (a SET-domain-containing histone methyltransferase) and JMJD6 (Rahman et al., 2011). The precise role of JMJD6 remains unclear; it was initially thought to be a histone arginine demethylase (Chang, Chen, Zhao, & Bruick, 2007) but more recently has been described as a hydroxylase involved in regulating RNA splicing (Webby et al., 2009). The bromodomain inhibitors would be predicted to act at the CpG low promoters and allow modulation of some of the most potent LPS responses that result in induction of IL-6, IL-12 and NO, which could have advantages in downregulating systemic inflammation during severe infections (Smale et al., 2014). The specific bromodomain inhibitor JQ1 has been successfully used in murine models of myeloma to modulate the function of the oncogene c-Myc (Delmore et al., 2011). This suggests that a more targeted approach using these agents is possible. In an alternative but related approach a synthetic inhibitor that interferes with the recognition of histone acetylation by the BET family of proteins, and thus the formation of the complex driving mRNA generation from the associated gene, was shown to be capable of modulating the response to LPS (Nicodeme et al., 2010). The inhibitor (I-BET), a synthetic histone mimic, reduced the induction of a number of inflammatory cytokines (including IL-6, Interferon-β, IL-1β) by activated murine macrophages following LPS stimulation. Furthermore, this effect was selective for inflammatory responses, as the expression of housekeeping genes was not modified. I-BET also demonstrated efficacy in reducing the death rate in mice following LPS challenge or bacterial sepsis and therefore holds promise as a novel approach regulating the inflammatory response. More recently, these results have been reproduced in primary human macrophages. The authors demonstrated that I-BET151 led to decreased IFN responses following TLR4 and TNF-α stimulations (Chan et al., 2015).

**Fig. 2:**
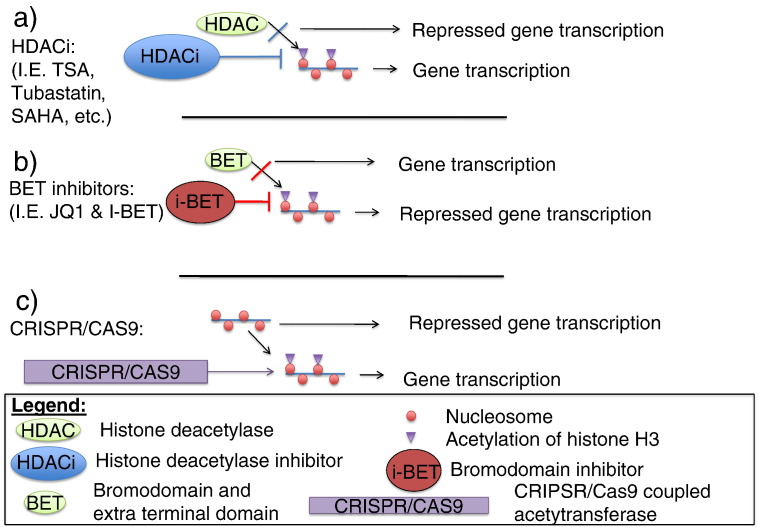
**a**) Schematic representation of the mode of action of HDACi. Leading to the maintenance of the acetyl post-translational modification and thereby ongoing gene transcription. **b**) Schematic representation of bromodomain inhibitors such as I-BET151 or JQ1, these inhibit the binding of bromodomains such as BRD4 to the underlying chromatin thereby inhibiting gene transcription. **c**) Schematic representation of the mode of action of CRISPR/CSAS9 construct, this leads to the acetylation of the promoter region of a specific gene leading to its transcription.

Recent advances in the field of clustered regularly interspaced short palindromic repeats (CRISPR) technology have enabled targeted modification of the epigenome in an effort to manipulate gene regulation (Fig. 2). The CRISPR–Cas9 editing system has enabled researchers to target a specific location in the genome, therefore enabling the detailed analysis of the function of specific epigenetic changes. The CRISPR–Cas9 systems are a more robust and high throughput approach, compared to previous methods (Ledford, 2015).

An inactive Cas9 (CRISPR-associated protein 9) was used as a programmable CRISPR/CAS9 construct fused with an acetyltransferase (p300) allowing the acetylation of the promoter region of specific genes (Hilton et al., 2015). The fusion protein catalyzes the acetylation of H3K27 at its target sites, leading to transcriptional activation of target genes. The results of this study supported targeted acetylation as a causal mechanism of transactivation (Hilton et al., 2015).

The CRISPR–Cas9 system has also been used to with a deactivated Cas9 enzyme fused to LSD1 (a histone demethylase), and subsequently programmed to target regions of DNA believed to enhance the expression of a range of genes (Kearns et al., 2015). The result was a functional map of genetic ‘enhancer’ sequences that provided further insight into enhancer regions and their genomic locations.

This ground-breaking development paves the way for a reversible editing of the epigenome and thereby a specific approach targeting cell function by influencing gene expression without altering the underlying DNA. This could lead to the targeted resolution of inflammatory processes or to the manipulation of individual epigenomes to reduce the susceptibility to disease or prevent the relapse of malignancies. Future work, focusing on a range of alternative chromatin modifiers and orthogonal dCas9 systems will allow researchers to perform even more complex epigenome engineering.

## 8 Conclusion

In conclusion, it is apparent that our increased understanding of how chromatin remodeling influences immune function and specialization has opened up the therapeutic potential of the area. Initial approaches to modulate systemic inflammation in sepsis and to reactivate HIV from latency show the potential of the approach. In addition the development of site specific and more potent HDACi, bromodomain inhibitors and CRISPR technology offer powerful tools with which to exploit the potential modulation of histone PTMs regulating gene transcription in the management of infectious diseases. Finally pathogens themselves may express enzymes to modify histone PTMs and regulate gene transcription. These may then provide potential therapeutic targets if selective inhibitors can be identified as evidenced by potential interest in inhibiting *P. falciparum* HDACs to limit parasite growth and development (Andrews et al., 2012). Thus modulation of histone PTM may offer a range of potential therapeutic options in the future.

## Conflict of interest statement

The authors declare that there are no conflict of interests.

## Funding sources

J. Cole is supported by a Wellcome Trust (104437/Z/14/Z) clinical research training fellowship.

D. H. Dockrell is supported by MRC (MR/M017931/1) and Wellcome Trust funding and has received research funding from GSK (COL100037467) and help with conference attendance, speaker or advisory board fees from ViiV, Gilead, BMS, JCG or MSD.

M. J. Dickman is supported by the Biotechnology and Biological Sciences Research Council UK (BB/M012166/1) and the Engineering and Physical Sciences Research Council UK.

The funding sources had no involvement in this work.

## Figures and Tables

**Table 1:** Host-pathogen interactions and histone post-translational modifications.

Organism	Mechanism	Enzyme	Histone PTM	Consequence	References
*Mycobacteria*					
*Mycobacterium tuberculosis*	Secreted mycobacterial protein Rv1988	Methyltransferase	Methylates histone H3 at arginine 42	Represses gene expression leading to decreased production of ROS, NOS and NADPH oxidase	Yaseen et al., 2015

*Bacteria*					
*Legionella pneumophilia*	Secretes RomA	Methyltransferase	Trimethylates lysine 14	Decreased immune gene transcription in particular IL-6 and TNF-α	Rolando et al., 2013
*Listeria monocytogenes*	Secretes lysteriolysin O and internalin B	Dephosphorylation	Deacetylation of lysine 18	Decreased immune gene transcription	Hamon et al., 2007
		Deacetylation via translocation of sirt2			Eskandarian et al., 2013
*Helicobacter pylori*	Unclear but involves cytotoxin-associated gene A pathogenicity island		Dephosphorylation of serine10, decreased acetylation of lysine 23	Increased inflammation and IL-8 production, and upregulation of oncogene c-Jun	Ding et al., 2010
*Escherichia coli*	LPS stimulation of TLR	Acetylation	Histone H3 lysine 14 and H4 lysine 8 hyperacetylation	Increased inflammation	Modak et al., 2012
*Shigella flexneri*	Injected OspF	Phospholyase	Phosphorylates heterochromatin Protein 1 γ at serine 83	Modulates gene expresion in particular IL-8 and cell proliferation	Harouz et al., 2014
*Anaplasma phagocytophilum*	Secretes ankyrin A	Deacetylation	Deacetylation of histone H3	Decrease in host defense genes including cytochrome B-245, beta polypeptide.	Garcia-Garcia et al., 2009

*Virus*					
Human cytomegalovirus	Regulation of DOT1L	Methyltransferase	Histone H3 K79 dimethylation and H4 lysine 16 deacetylation	Decreased gene expression	O'Connor, DiMaggio, Shenk, & Garcia et al., 2014
HIV	HIVssRNA	TLR8 mediated	Histone H4 acetylation and H3 lysine 4 trimethylation, decrease in lysine 27 trimethylation	TNF-α release	Han et al., 2012
Human adenovirus	Early region 1a	Deacetylation	Histone H3 lysine 18	Oncogenic transformation	Oh, Traktman, & Knipe, 2015
Influenza A virus	Secretes NS1 histone like mimic		Cytosolic signaling human PAF1 transcription elongation complex	Reduces antiviral gene expression	Marazzi et al., 2012

**Table 2:** Clinical trials of HDACi in HIV eradication.

Category	Name	Clinical Trial ID	Status/estimated completion date	Details	Sponsor
HDACi	Valproic acid	NCT00614458	Terminated (September 2008)	10,493 — MK-0518 intensification and HDAC inhibition in depletion of resting CD4 + T-cell HIV infection	University of North Carolina, Chapel Hill
HDACi	Romidepsin	NCT01933594	Recruiting/May 2016	Phase I/II trial; evaluating the safety and efficacy of single-dose romidepsin in combination with ART in HIV-1 infected adults with suppressed viral load	National Institute of Allergy and Infectious Diseases (NIAID)
HDACi	Romidepsin	NCT02092116	Ongoing, recruitment closed/December 2015	An open phase I/IIa study to evaluate the safety and effict of therapeutic HIV-1 immunization using vacc-4 × + rhuGM-CSF, and reactivation using romidepsin on the viral reservoir in virologically suppresed HIV-1 infected adults on ART. (REDUC)	Bionor Immuno AS
HDACi	Panobinostat	NCT01680094	Completed (January 2014)	The safety and efficacy of the HDACi panobinostat for purging HIV-1 for the latent reservoir (CLEAR) study	University of Aarhus
HDACi	Panobinostat	NCT02471430	To start (September 2015)/February 2020	A phase II pilot study to assess the safety and efficacy of combined treatment with pegylated interferon-alpha2a and the HDACi panobinostat for reducing the residual reservoir of HIV-1 infected cells in ART-treated HIV-1 positive individuals (ACTIVATE).	Massachusetts General Hospital
HDACi	Vorinostat	NCT01319383	In progress/March 2016	A phase I/II investigation of the effect of vorinostat on HIV RNA expression in resting CD4 + T-cells of HIV-infected patients receiving stable ART	University of North Carolina, Chapel Hill
HDACi	Vorinostat	NCT02336074	To start (September 2015)/July 2018	Research in viral eradication of HIV reservoirs (RIVER); prospective RCT comparing raltegravir boosted HAART with or without ChAd prime + MVA boost HIV vaccine + 28 day course of vorinostat	Imperial College London
HDACi	Vorinostat	NCT02475915	Recruiting/April 2016	A randomized study to compare efficacy of vorinostat/hydroxychloroquine/maraviroc (VHM) in controlling HIV after treatment interruptions in subjects who initiated ART during acute HIV infection.	South East Asia Research Collaboration with Hawaii
